# Justification: gain or game

**DOI:** 10.1590/0100-3984.2023.0117

**Published:** 2024-05-07

**Authors:** Mannudeep K. Kalra, Mônica Oliveira Bernardo, Lina Karout, Alair Augusto Sarmet Moreira Damas dos Santos

**Affiliations:** 1 Department of Radiology, Massachusetts General Hospital, Harvard Medical School, Boston, MA, USA; 2 Hospital Miguel Soeiro, Pontifícia Universidade Católica de São Paulo (PUC-SP), Sorocaba, SP, Brazil; 3 Departamento de Radiologia, Faculdade de Medicina da Universidade Federal Fluminense (UFF), Niterói, RJ, Brazil

## INTRODUCTION

Innovations and access to medical imaging have increased its utilization. While some regard imaging as the modern stethoscope to peer within the opaque anatomy and complex physiology, others caution over radiation risks, the workup required when there are incidental findings, and the healthcare costs related to over-testing. Although the justified use of imaging provides invaluable information on the presence and extent of abnormalities, the statistics on unjustified procedures should not be ignored.

### Digitalizing justification

**Figure 1:**
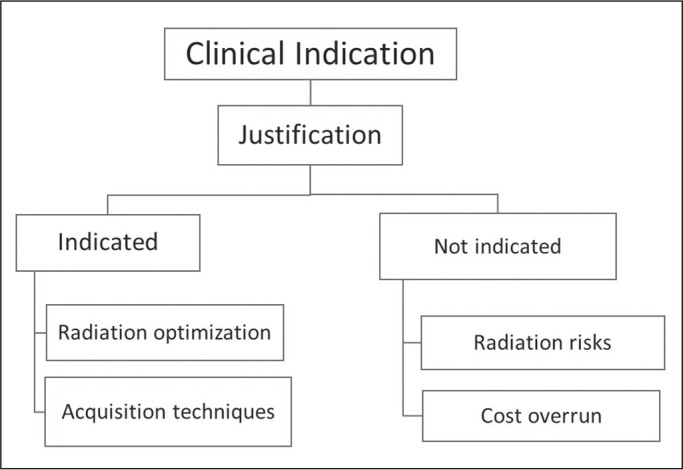
Justification helps determine whether or not an ordered imaging test is truly indicated. Non-indicated tests entail unnecessary radiation risks and cost overruns. The indications that help determine justification can also help optimize acquisition techniques and radiation doses.

Stemming from concerns over spiraling costs, referral guidelines and appropriateness criteria to promote the justification of imaging tests have been proposed by various organizations^(1-3)^. With evidence-based findings and consensus statements by multidisciplinary committees of expert physicians, these guidelines recommend specific imaging pathways or review the risks, benefits, and individual utility of imaging tests for several dozen common clinical indications (Figure 1). The American College of Radiology Appropriateness Criteria, incorporated within the electronic health records (EHRs), help physicians determine the appropriateness of imaging tests based on certain symptoms, signs, and diagnoses^(1)^. The EuroSafe Referral Guidelines for Imaging provide online clinical decision support (CDS) systems for several clinical indications and imaging modalities^(2)^. Those CDS systems provide the utility of different imaging examinations and symbols for the relative radiation levels (a single radiation icon for low-dose radiography versus multiple icons for higher-dose computed tomography). Some provide the relative costs of imaging tests with USD/Euro symbols.

At Mass General Brigham, the EHR-embedded CDS system lets the referring physician select a convenient site (hospital versus offsite locations), day, and time for an imaging test^(3)^. In addition to the convenience, the electronic CDS system allows referral practices to be audited and monitored over time.

### Limitations

Like legal justice, which is an absolute necessity but not always fair or timely, justification has its limitations. Prior studies have documented a decline in the utilization of imaging tests after the implementation of a CDS system^(4-7)^. Weilburg et al. reported a 28% decline in the use of high-cost imaging tests from 2007 to 2013, after a utilization management program was instituted in an outpatient setting^(6)^. However, some studies have suggested that those initial reductions in imaging utilization are temporary^(8,9)^. Other investigations have raised concerns about the substantial lack of consistency between the clinical indications specified in the CDS system and the symptoms, signs, and diagnoses described in the EHRs^(10)^. Gupta et al. found a 4.2% error rate in CDS system orders for computed tomography pulmonary angiography^(10)^. Such inconsistencies do not necessarily suggest malicious intent and might stem from errors in the EHR, a complex clinical presentation, or patient expectations or demands. The Brazilian College of Radiology and Diagnostic Imaging has initiated efforts to create referral guidelines for imaging in Brazil.

### Future

The disconcerting frequency of incorrect and incomplete clinical information in CDS systems offers an opportunity to aid or automate the determination of the best imaging tests based on the text recorded in the EHR^(11,12)^. Gish et al. reported that the proportion of referring physicians who preferred the imaging tests suggested by a new free-text-based artificial intelligence (AI) tool was significantly greater than that of those who preferred the traditional order-entry CDS system (58.9% vs. 41.1%; *p* < 0.01)^(11)^. In addition, the free-text-based AI tool predicted orders correctly in 91.7% of cases. Another study, conducted by Ramgopal et al., demonstrated that an AI tool can accurately predict the need for clinical and imaging tests in children presenting to the emergency department, with an area under the receiver operating characteristic curve of 0.89-0.99^(13)^. Given the excitement over Chat GPT, could the use of large language models further improve compliance or automated selection of imaging pathways? Only time and further studies will tell us if such a path leads to justice in justification.

## CONCLUSION

Implementing and monitoring justification in imaging are complex tasks. There are definite gains to be made from the justified use of imaging tests, including reductions in the associated risks and costs. However, the gamut of clinical presentations, physician practices, and patient preferences makes this a complicated game in search of a satisfactory solution.
